# *McrA* primers for the detection and quantification of the anaerobic archaeal methanotroph ‘*Candidatus* Methanoperedens nitroreducens’

**DOI:** 10.1007/s00253-016-8065-8

**Published:** 2017-01-13

**Authors:** Annika Vaksmaa, Mike S. M. Jetten, Katharina F. Ettwig, Claudia Lüke

**Affiliations:** 1 Department of Microbiology, IWWR, Radboud University Nijmegen, Nijmegen, The Netherlands; 2 Department of Biotechnology, Delft University of Technology, Delft, The Netherlands; 3 Soehngen Institute of Anaerobic Microbiology, Nijmegen, The Netherlands

**Keywords:** ‘*Candidatus* Methanoperedens nitroreducens’, Anaerobic oxidation of methane, ANME, *mcrA*

## Abstract

The nitrogen and methane cycles are important biogeochemical processes. Recently, ‘*Candidatus* Methanoperedens nitroreducens,’ archaea that catalyze nitrate-dependent anaerobic oxidation of methane (AOM), were enriched, and their genomes were analyzed. Diagnostic molecular tools for the sensitive detection of ‘*Candidatus* M. nitroreducens’ are not yet available. Here, we report the design of two novel *mcrA* primer combinations that specifically target the alpha sub-unit of the methyl-coenzyme M reductase (*mcrA*) gene of ‘*Candidatus* M. nitroreducens’. The first primer pair produces a fragment of 186-bp that can be used to quantify ‘*Candidatus* M. nitroreducens’ cells, whereas the second primer pair yields an 1191-bp amplicon that is with sufficient length and well suited for more detailed phylogenetic analyses. Six different environmental samples were evaluated with the new qPCR primer pair, and the abundances were compared with those determined using primers for the 16S rRNA gene. The qPCR results indicated that the number of copies of the ‘*Candidatus* M. nitroreducens’ *mcrA* gene was highest in rice field soil, with 5.6 ± 0.8 × 10^6^ copies g^−1^ wet weight, whereas Indonesian river sediment had only 4.6 ± 2.7 × 10^2^ copies g^−1^ wet weight. In addition to freshwater environments, sequences were also detected in marine sediment of the North Sea, which contained approximately 2.5 ± 0.7 × 10^4^ copies g^−1^ wet weight. Phylogenetic analysis revealed that the amplified 1191-bp *mcrA* gene sequences from the different environments all clustered together with available genome sequences of *mcrA* from known ‘*Candidatus* M. nitroreducens’ archaea. Taken together, these results demonstrate the validity and utility of the new primers for the quantitative and sensitive detection of the *mcrA* gene sequences of these important nitrate-dependent AOM archaea. Furthermore, the newly obtained *mcrA* sequences will contribute to greater phylogenetic resolution of ‘*Candidatus* M. nitroreducens’ sequences, which have been only poorly captured by general methanogenic *mcrA* primers.

**Electronic supplementary material:**

The online version of this article (doi:10.1007/s00253-016-8065-8) contains supplementary material, which is available to authorized users.

## Introduction

Methane is an important greenhouse gas (GHG) that contributes approximately 20% to global warming (Myhre et al. 2013). Since the advent of industrialization, atmospheric concentrations of methane have increased by 150%, potentially further exacerbating climate change (Schwietzke et al. 2016). Evaluating the contribution of environmental microorganisms that produce or consume this significant GHG is essential for understanding methane sources and sinks and developing mitigation strategies for methane released into the atmosphere. Most research on microorganisms involved in the methane cycle has focused on aerobic methanotrophic bacteria that inhabit oxic environments or archaea that produce methane in anoxic zones. However, recent studies have revealed that in the anoxic layers of soils and sediments, methane is consumed by anaerobic methanotrophic bacteria and/or archaea that use alternate electron acceptors such as nitrite, nitrate, or iron (Egger et al. 2015; Ettwig et al. 2010; Raghoebarsing et al. 2006).

Enrichment cultures inoculated with freshwater sediment exhibited coupling of the reduction of nitrite to the anaerobic oxidation of methane (Ettwig et al. 2008; Raghoebarsing et al. 2006). The corresponding nitrite-dependent methanotrophic bacteria were identified as belonging to the bacterial NC10 phylum and named ‘*Candidatus* Methylomirabilis oxyfera’ (Ettwig et al. 2010). This microorganism exhibits an intra-aerobic metabolism in which nitric oxide is hypothesized to be dismutated to oxygen and nitrogen gas. The oxygen could subsequently be used by the canonical particulate methane monooxygenase encoded by *pmoCAB*.

Archaea that oxidize methane anaerobically were initially discovered in marine environments, where they carry out sulfate-dependent anaerobic oxidation of methane (S-AOM). These anaerobic methane-oxidizing archaea (ANME) have been estimated to oxidize up to 90% of released methane before it reaches the atmosphere (Hinrichs and Boetius 2002; Knittel and Boetius 2009). ANMEs are divided into three lineages, ANME-1, ANME-2, and ANME-3 (Knittel et al. 2005; Nauhaus et al. 2005; Stadnitskaia et al. 2005) and are further divided into sub-clades in some cases. All three lineages have been detected in marine and freshwater environments.

Recently, the genomes of ANME-2d archaea enriched in bioreactors fed with methane, nitrate, and ammonium or methane and nitrate were obtained (Arshad et al. 2015; Haroon et al. 2013). These Euryarchaea, which are capable of coupling nitrate reduction to anaerobic methane oxidation, were identified as ‘*Candidatus* Methanoperedens nitroreducens.’ Phylogenetic analysis revealed that these archaea are related to *Methanosarcina* in the Methanosarcinales order (Haroon et al. 2013) and are classified as GOM Arc I in the ribosomal RNA (rRNA) SILVA database. The GOM Arc I consists of the ANME-2d group as well as the original GOM Arc I group with sequences from the Gulf of Mexico (Mills et al. 2003).

‘*Candidatus* M. nitroreducens’ possesses all genes of the (reverse) methanogenic pathway (Arshad et al. 2015; Haroon et al. 2013). The best-characterized enzyme of methanogenesis and AOM is methyl-coenzyme M reductase (MCR). In methanogenesis, MCR catalyzes the terminal step of the pathway, resulting in the release of methane. In the anaerobic oxidation of methane, MCR functions in a reverse mode (Hallam et al. 2003, 2004; Krüger et al. 2003), catalyzing the activation of methane (Krüger et al. 2003). The genomes of two ‘*Candidatus* M. nitroreducens’ strains have been assembled and analyzed. In both genome assemblies, the complete reverse methanogenesis pathway including the *mcrABCDG* genes was identified (Arshad et al. 2015; Haroon et al. 2013), and the genomes contained only a single copy of the 16S rRNA and the *mcrA* gene. Furthermore, the enzymes for nitrate reduction to nitrite and nitrite reduction to ammonium appeared to be encoded by *narGH*- and *nrf*-type genes, respectively (Arshad et al. 2015).

For ‘*Candidatus* M. oxyfera’ bacteria, specific primers for both the 16S rRNA gene and the *pmoA* gene have been designed (Ettwig et al. 2009; Luesken et al. 2011). Analyses of various environmental samples using these primers have demonstrated that ‘*Candidatus* M. oxyfera’ is present in peat lands, lake sediments, wastewater treatment systems, rice fields, and various other anoxic environments (Deutzmann and Schink 2011; Hu et al. 2014; Zhou et al. 2014; Zhu et al. 2012). As nitrate concentrations in freshwater environments are generally higher than those of nitrite or sulfate, ‘*Candidatus* M. nitroreducens’ may contribute significantly to nitrate-dependent AOM in these environments (Vaksmaa et al. 2016). To detect ‘*Candidatus* M. nitroreducens’ in environmental samples, specific fluorescence in situ hybridization (FISH) probes have been designed (Schubert et al. 2011). The development of quantitative detection methods based on the 16S rRNA gene has also been reported (Ding et al. 2015).

Although the 16S rRNA gene is most commonly used for phylogenetic surveys, the *mcrA* gene is an alternative and more specific biomarker for the detection of methanogens and ANMEs in the environment. Although previously published *mcrA* primers were designed to mainly target all known methanogens and ANMEs, most have a strong bias toward certain methanogens or specific groups of ANMEs (Hales et al. 1996; Juottonen et al. 2006; Luton et al. 2002; Nunoura et al. 2008). Available general *mcrA* primers are not well suited to capturing *mcrA* sequences of ‘*Candidatus* M. nitroreducens’ in the environment, potentially resulting in underrepresentation in molecular surveys. Furthermore, differentiating between phylogenetically closely related methanogens and methanotrophs is crucial to directly link observed diversity with the organisms responsible for either methane oxidation or methane production. In the current study, we developed two novel *mcrA* primer pairs that specifically target ‘*Candidatus* M. nitroreducens’ for use in quantification and more refined phylogenetic analysis. We used these primers to study the distribution and abundance of ‘*Candidatus* M. nitroreducens’ in various ecosystems. For comparison, we validated the use of 16S rRNA gene probes designed for FISH analysis as qPCR primers and compared the results with the diversity and abundance obtained with the novel *mcrA* primers.

## Materials and methods

### Environmental samples

Environmental samples were obtained from six different locations: rice field soils (Vercelli, Italy), sludge from a brewery wastewater treatment plant (Lieshout, The Netherlands), North Sea sediment (The Netherlands), polluted Citarum River sediment (Indonesia), Jordan River sediment (UT, USA), and State Channel sediment (UT, USA). In addition to the environmental samples, an enrichment culture (AOM enrichment Vercelli) of ‘*Candidatus* M. nitroreducens’ was used as a sample for primer validation (Vaksmaa et al in preparation). The samples were stored at −20 °C prior to DNA extraction. Detailed information on the geographic locations is presented in Table S1.

### Primer design, DNA extraction, and PCR amplification

For primer design, 20,000 high-quality *mcrA* sequences deposited in the NCBI GenBank database (Benson et al. 2013) were downloaded and aligned, and the lengths of these sequences were inspected. From the alignment of 20,000 *mcrA* sequences, 45 available full-length *mcrA* sequences (two belonging to ‘*Candidatus* Methanoperedens nitroreducens’) were used for primer design using the probe design tool implemented in ARB (Ludwig et al. 2004). The designed *mcrA* primer set McrA159F/McrA345R amplifies a 186-bp fragment and has a predicted annealing temperature of 62 °C. The McrA169F/McrA1360R primer pair yields a 1191-bp fragment. Detailed information on the *mcrA* primers and 16S rRNA primers used in this study is provided in Table 1. Commonly used general *mcrA* gene primers were in silico evaluated for their ability to target ‘*Candidatus* Methanoperedens nitroreducens,’ and the number of mismatches is brought out in Table 2. For comparison, the 16S rRNA gene of ‘*Candidatus* Methanoperedens nitroreducens’ was targeted with the clade-specific primers AAA641F and AAA834R (previously reported as FISH probes) (Schubert et al. 2011). These primers amplify a 212-bp fragment with an optimal annealing temperature of 60 °C. DNA was extracted from all samples with the PowerSoil® DNA Isolation Kit. First, 0.1–0.35 g of soil was weighed into the 2-ml tubes provided with the kit, which contained buffer and beads. The following steps were performed according to the manufacturer’s protocol (MO BIO Laboratories Inc., Carlsbad, USA). DNA quantity was assessed using a microspectrophotometer (NanoDrop, ND-1000, Isogen Life Science, The Netherlands). All PCR reactions were performed using PerfeCTa Quanta master mix (Quanta Biosciences, Gaithersburg, USA) with the following composition: 1 μl each of 20 μM of the forward and reverse primers, 12.5 μl of PCR master mix and 9.5 μl of Milli-Q water. The PCR temperature gradient program was 96 °C for 5 min, followed by 45 cycles of 96 °C for 30 s, gradient (55–68 °C) for 45 s, and 72 °C for 45 s and a final extension at 72 °C for 10 min.

**Table 1:** List of PCR and qPCR primers used for mcrA and 16S rRNA gene amplification

Primer name	Sequence 5′–3’	Nr of bases	Primer binding site 5′ to 3′	Tm (°C)	GC (%)	Product size (bp)
McrA159F	AAAGTGCGGAGCAGCAATCACC	22	159–181	66.5	55	186
McrA345R	TCGTCCCATTCCTGCTGCATTGC	23	322–345	71	57	
McrA169F	GCA GCA ATC ACC AAG AAG AGA GG	23	169–192	59.9	52	1191
McrA1360R	TGCCTCTTTGTGGAGGTACATGGA	24	1336–1360	65.6	50	
16S rRNA AAA641F	ACTGDTAGGCTTGGGACC	17	576–593	51.4	59	193
16S rRNA AAA834R	ATGCGGTCGCACCGCACCTG	20	768–788	72.6	70	

Primer binding site refers to the nucleotide position at the *mcrA* gene of ‘*Candidatus* M. nitroreducens’

### Cloning, sequencing, and phylogenetic analysis

The sizes of the PCR products obtained with the McrA159F/McrA345R, McrA169F/McrA1360R, or AAA641F/AAA834R primer pairs were evaluated by gel electrophoresis on 1% agarose gels. The fragments were purified using the GeneJET PCR purification kit according to the manufacturer’s protocol (Thermo Scientific, Landsmeer, The Netherlands). The amplified PCR products were cloned using the pGEM-T Easy cloning vector (Promega, USA) and used to transform *E. coli* XL1 Blue competent cells. The cells were plated on Luria-Bertani (LB) agar plates containing 20 μl of 100 mg/ml ampicillin, 35 μl of 2% X-Gal, and 35 μl of 100 mM IPTG. The plates were incubated at 37 °C overnight. Colony PCR was performed by direct PCR using the M13F and M13R primers. The PCR program consisted of initialization at 96 °C for 10 min, followed by 40 cycles of amplification at 96 °C for 45 s, 57 °C for 30 s, and 72 °C for 30 s and a final elongation step at 72 °C for 5 min. The colonies resulting in amplification of a fragment of the correct size were grown in 5 ml of LB medium overnight at 37 °C prior to plasmid isolation with a GeneJET Plasmid Miniprep Kit (Thermo Scientific, The Netherlands). The inserts were sequenced at BaseClear B.V. (Leiden, Netherlands) or Macrogen (Amsterdam, Netherlands). For short fragments, the MF primer (5′TTTCCCAGTCACGACGTTG′3) was used, and to retrieve longer fragments, sequencing was also performed with the MR primer (5′GGATAACAATTTCACACAGG′3). The quality of the sequences was assessed with the Chromas Lite 2.01 (Technelysium Pty Ltd., Australia) software. All DNA sequences were imported into the *mcrA* ARB database. ARB version 5.5 was used for phylogenetic comparison (Ludwig et al. 2004). Phylogenetic trees based on the DNA sequences were calculated using the neighbor-joining algorithm with the Jukes-Cantor correction. Sequences were further analyzed by BLASTn and BLASTx at NCBI (Altschul et al. 1990).

### Quantification by qPCR

The *mcrA* and 16S rRNA gene copy numbers in the environmental samples were quantified with the primer set McrA159F/McrA345R and the 16S rRNA gene primers AAA641F/AAA834R. All qPCR reactions were performed using PerfeCTa Quanta master mix (Quanta Biosciences, Gaithersburg, USA) and 96-well optical plates (Bio-Rad Laboratories, Hercules, England). Each reaction was performed in triplicate on duplicate DNA extractions. All reactions were performed using the Bio-Rad IQ™ 5 cycler (Biorad, USA). Negative controls were added to each plate by replacing the sample volume with autoclaved Milli-Q water. Standard curves were constructed by tenfold serial dilution of a known copy number of the pGEM-T easy plasmid with inserted DNA of the target gene.

### In silico evaluation of 16S rRNA primers

The specificity and intra-group coverage of the 16S rRNA gene primers DP397F/DP569R (Ding et al. 2015) and the primers AAA641F/AAA834R, which target ‘*Candidatus* M. nitroreducens,’ were evaluated. The comparison was carried out in ARB (Ludwig et al. 2004) using the GOM Arc I group as a representative group for ‘*Candidatus* M. nitroreducens’ and related sequences. The specificity and intra-group coverage of both primer sets were evaluated using the non-redundant version of the SILVA SSU Ref dataset (release 119; (Quast et al. 2013)), which contains 535,004 high-quality 16S rRNA gene sequences, of which 109 belong to GOM Arc I.

### Nucleotide sequence accession numbers

Representative sequences were deposited at GenBank under the accession numbers KX290067–KX290105 for *mcrA* sequences amplified with the McrA159F/McrA345R primers and under the accession numbers KX290017–KX290044 for *mcrA* sequences obtained with the primers McrA169F/McrA1360R. The 16S rRNA gene sequences were deposited under accession numbers KX290045–KX290065.

## Results

### Specificity of the novel *mcrA* primers for qPCR

**Fig. 1:**
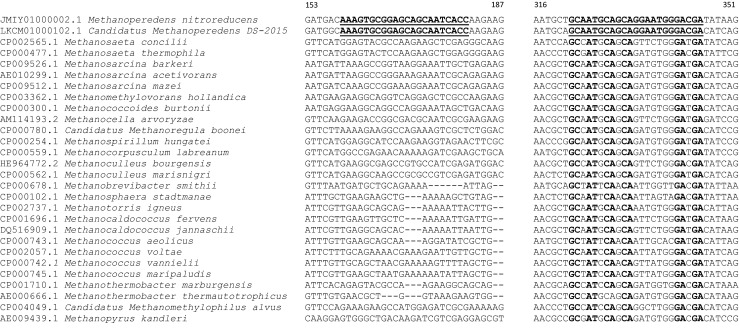
Excerpt of the full-length sequence alignment of the *mcrA* genes of anaerobic methanotrophs and methanogens. The binding sites and conserved positions of the McrA159F forward and McrA345R reverse primers are indicated in *2*

To design an *mcrA* primer set specific for ‘*Candidatus* M. nitroreducens,’ available full-length *mcrA* sequences (45 sequences) covering the diversity of known methanotrophs and methanogens were aligned and used for primer design. Representative sequences and the primer-binding positions are depicted in Fig. 1. Two sites at an appropriate distance for qPCR amplification (at nucleotide positions 159–181 and 322–345, respectively) were conserved between the two ‘*Candidatus* M. nitroreducens’ sequences but were different in all other archaeal *mcrA* sequences. The resulting McrA159F/McrA345R primer pair amplifies a fragment of 186 bp, suitable for qPCR. The forward primer McrA159F possesses four mismatches with the *mcrA* sequence of the closest methanogen, *Methanobacterium alcaliphilum.* The reverse primer McrA345R possesses three mismatches with the *mcrA* sequences of the methanogens *Methanothermobacter wolfeii* and *Methanohalophilus halophilus*. The optimal annealing temperature of 62 °C was determined by gradient PCR using DNA extracted from rice field soil. All 40 PCR products amplified from DNA from the environmental samples and the enrichment culture were cloned and sequenced and corresponded to the expected part of the *mcrA* gene. The sequencing resulted in five to seven clone sequences per each environmental sample. All of the sequences had very high similarity to the *mcrA* gene of the two described ‘*Candidatus* M. nitroreducens’ strains (91–100% at the nucleotide level and 97–100% at the amino acid level (Table S2)).

**Table 2:** Overview of mcrA primers commonly used to amplify methanogenic and methanotrophic communities

Author	Primer name	Nr of bp	Binding position	Primer sequence 5′–3′	Mismatches
Luton et al. (2002)	ML-F	32	1021–1053	GGTGGTGTMGGATTCACACARTAYGCWACAGC	6
	ML-R	23	1468–1491	TTCATTGCRTAGTTWGGRTAGTT	3
Springer (1995)	McrAF	17	988–1005	TAYGAYCARATHTGGYT	5
	McrAR	17	1477–1491	ACRTTCATNGCRTARTT	4
Hales et al. (1996)	ME1F	20	727–747	GCMATGCARATHGGWATGTC	6
	ME2R	21	1469–1490	TCATKGCRTAGTTDGGRTAGT	4
	ME3F	20	1036–1056	GGTGGHGTMGGWTTCACACA	5
Nunoura et al. (2006)	Type c–d ANME-2	24	984–1008	GCTCTACGACCAGATMTGGCTTGG	3
		25	1058–1083	CCGTAGTACGTGAAGTCATCCAGCA	9
Nunoura et al. (2006)	Type e	25	1220–1245	CHCTGGAAGATCACTTCGGTGGTTC	5
		24	1363–1387	RTATCCGAAGAARCCSAGTCKRCC	5
Nunoura et al. (2006)	Type a-b ANME-1	20	1000–1020	TGGTTCGGAACGTACATGTC	4
		20	1562–1582	TCTYYTCCAGRATGTCCATG	6
Nunoura et al. (2008)	ME3MF	23	1015–1038	ATGTCNGGTGGHGTMGGSTTYAC	5
	ME3MF-3	23	1015–1038	ATGAGCGGTGGTGTCGGTTTCAC	6
Present study	McrA 159F	22	159–181	AAAGTGCGGAGCAGCAATCACC	0
Present study	McrA 345R	23	322–345	TCGTCCCATTCCTGCTGCATTGC	0

The number of mismatches to the ‘*Candidatus* M. nitroreducens’ mcrA sequences is indicated

### qPCR quantification of ‘*Candidatus* Methanoperedens nitroreducens’ *mcrA* and 16S rRNA gene copies in environmental samples

The newly designed *mcrA* primers McrA159F/McrA345R were used with DNA extracted from six environmental samples. In addition, the results were compared with the copy numbers obtained with the primers AAA641F/AAA834R targeting the 16S rRNA gene of ‘*Candidatus* M. nitroreducens.’

Two 16S rRNA primer sets, the primer pairs DP397F/DP569R (Ding et al. 2015) and AAA641F/AAA834R, have been proposed to target ‘*Candidatus* M. nitroreducens’ and the GOM Arc I group, respectively. Here, we analyzed the applicability of these primer sets in silico as specific qPCR primers to target the GOM Arc I group. The intra-group coverage and the number of out-group targets with one to three allowed mismatches are presented in Table 3. The primer pair AAA641F/AAA834R exhibited higher intra-group coverage (65–84%) than the DP397F/DP569R primers, which covered less than 60% of the GOM Arc I sequences at zero mismatch. Thus, we experimentally tested the AAA641F/AAA834R primers using DNA from the environmental samples and the enrichment culture and sequenced the PCR products. Twenty-one of the resultant clone sequences were highly similar to the 16S rRNA gene sequences of the two described ‘*Candidatus* M. nitroreducens’ strains, whereas two clone sequences did not correspond to ‘*Candidatus* M. nitroreducens’ (Table S3).

**Table 3:** The specificity and fidelity of previously described 16S rRNA gene primers for the total GOM Arc I group

	Intra-group coverage of GOM Arc I (%)				Hits in non-GOM Arc I			
	Mismatches				Mismatches			
	0	1	2	3+	0	1	2	3+
DP397F	23	64	79	85	*3*	*8*	*249*	2221
DP569R	59	*83*	*84*	*85*	*1*	44	343	*1737*
AAA641F	*71*	*89*	*92*	*92*	21	616	3184	9751
AAA834R	*65*	78	80	84	7	*7*	*26*	*76*

The intra-group coverage and the number of non-target hits are shown with up to three mismatches. The highest intra-group coverage and the lowest number of out-group targets per primer are marked in italics

**Fig. 2:**
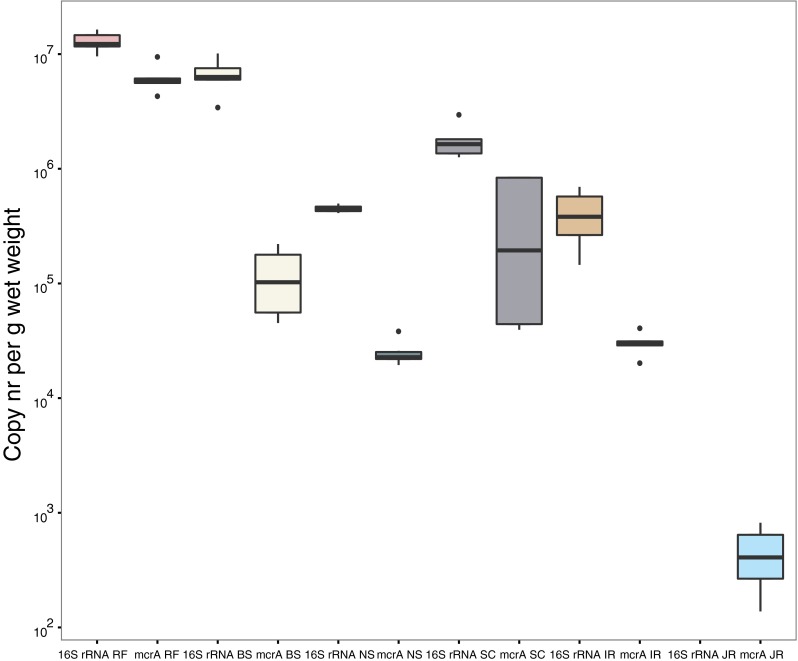
Boxplot depicting the abundance of ‘*Candidatus* M. nitroreducens’ in environmental samples as assessed by quantitative PCR of the 16S rRNA gene and *mcrA* gene. For each sample, six independent qPCR reactions of two DNA samples were performed. The environmental samples originated from rice field soil (RF), wastewater treatment plant sludge (BS), North Sea sediment (NS), State Channel sediment (SC), Indonesian river sediment (IR), and Jordan River sediment (JR). The *horizontal line* within each *box* represents the median, and the *error bars* represent the standard deviation. The *upper* and *lower* in each *box lines* represent the 75 and 25 percentiles, respectively. For the Jordan River sediment, no amplification was detected with Methanoperedens-specific 16S rRNA gene primers

In the qPCR analysis, the highest ‘*Candidatus* M. nitroreducens’ copy numbers were obtained in rice field soil, with an average *mcrA* gene copy number of 5.6 ± 0.8 × 10^6^ copies g^−1^ wet weight and an average 16S rRNA gene abundance of 1.3 ± 0.3 × 10^8^ copies g^−1^ wet weight. Rice field soil was followed by river sediment (State Channel, USA; 4.4 ± 4.4 × 10^5^ *mcrA* gene copies g^−1^ wet weight and 1.8 ± 0.6 × 10^7^ 16S rRNA gene copies g^−1^ wet weight), wastewater treatment plant sludge (1.2 ± 0.8 × 10^5^ *mcrA* gene copies g^−1^ wet weight and 6.7 ± 2.2 × 10^7^ 16S rRNA gene copies g^−1^ wet weight), Indonesian river sediment (3.0 ± 0.7 × 10^4^ *mcrA* gene copies g^−1^ wet weight and 4.2 ± 2.2 × 10^6^ 16S rRNA gene copies g^−1^ wet weight), and North Sea sediment (2.5 ± 0.7 × 10^4^ *mcrA* gene copies g^−1^ wet weight and 4.5 ± 0.3 × 10^6^ 16S rRNA gene copies g^−1^ wet weight). The lowest abundance was recorded in the sediment of the Jordan River (UT, USA), where qPCR did not result in any 16S rRNA gene amplification and only 4.6 ± 2.7 × 10^2^ copies of the *mcrA* gene g^−1^ wet weight were detected (Fig. 2).

### Phylogenetic analysis

**Fig. 3:**
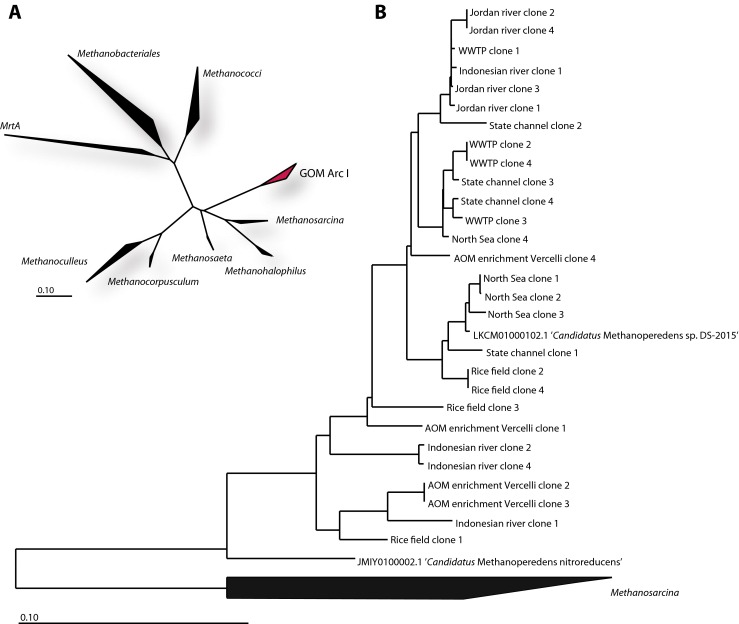
**a** Phylogenetic overview of methanogenic and anaerobic methanotrophic archaea based on *mcrA* gene sequences. The phylogenetic position of GOM Arc I archaea is marked in *pink*. **b** Phylogenetic tree of ‘*Candidatus* M. nitroreducens’ *mcrA* clone sequences (*n* = 28, 1191 bp). The tree includes the clones derived from this study as well as reference sequences of ‘ *Candidatus* Methanoperedens nitroreducens ANME-2d’ (GenBank accession number JMY01000002.1) and ‘*Candidatus* Methanoperedens sp. DS-2015’ (GenBank accession number LKCM01000080.1). The tree was computed using the neighbor-joining algorithm with the Jukes-Cantor correction (Color figure online)

In addition to the qPCR primers McrA159F/McrA345R, a second primer set was designed to amplify longer *mcrA* fragments. Conserved regions were identified at nucleotide positions 169–192 and 1336–1360. The resulting primer set, McrA169F/McrA1360R, amplifies a fragment of 1191 bp, suitable for detailed phylogenetic analysis. The primers were again tested using DNA extracted from the environmental samples and the enrichment culture as described in the “Materials and methods” section. Amplification resulted in a single band of the expected size, and sequence analysis indicated that all 40 sequences were highly similar to ‘*Candidatus* M. nitroreducens.’ The phylogenetic positions of these clones are depicted in Fig. 3. Clustering of sequences from the same environment was not observed, although all sequences clustered more closely with ‘*Candidatus* Methanoperedens sp. DS-2015’ than ‘*Candidatus* Methanoperedens nitroreducens ANME-2d.’ On average, the sequences exhibited higher identity to ‘*Candidatus* Methanoperedens sp. DS-2015’ (87–99% nucleotide sequence identity) than to ‘*Candidatus* Methanoperedens nitroreducens ANME-2d’ (85–90% nucleotide sequence identity). The sequence identities of all clones to the two described strains are provided in Table S4.

## Discussion

In this study, we developed specific and sensitive molecular detection tools to target nitrate-dependent anaerobic methanotrophic ‘*Candidatus* M. nitroreducens’ archaea. We designed two novel PCR primer sets for the *mcrA* gene of ‘*Candidatus* M. nitroreducens,’ thus providing a straightforward detection and quantification method. The primer set McrA159F/McrA345R results in the amplification of a 186-bp fragment and is suitable for quantification of *mcrA* gene copies by qPCR. The other primer set, McrA169F/McrA1360R, results in the amplification of a 1191-bp fragment that can be used in more accurate and detailed phylogenetic analyses.

The genomes of known ‘*Candidatus* M. nitroreducens’ strains possess only a single copy of the 16S rRNA gene and the *mcrA* gene, although copy numbers might differ for non-cultivated species. However, the copy numbers in the environmental samples obtained with the 16S rRNA gene primers were approximately two orders of magnitude higher than the copy numbers obtained with the *mcrA* primers. The newly designed *mcrA* primers are highly specific, whereas the 16S rRNA gene primers used in this study have the potential to amplify sequences from the whole GOM Arc I clade, possibly capturing a larger diversity of sequences that are less related to ‘*Candidatus* M. nitroreducens’. The target specificity was reflected in the sequence diversity: the sequenced PCR products obtained with the qPCR primer combination McrA159F/McrA345R all corresponded to the ‘*Candidatus* M. nitroreducens’ *mcrA* gene (97–100% identity at the amino acid level), whereas the sequenced PCR products of the 16S rRNA gene also included sequences (9%) that could be identified as closely related methanogens. This difference in specificity further suggests that the results obtained with these 16S rRNA gene PCR primers may overestimate the copy numbers of ‘*Candidatus* M. nitroreducens’ in the environment. Overall, the *mcrA* primers were more specific, and qPCR quantification of *mcrA* copy numbers may more accurately reflect the number of ‘*Candidatus* M. nitroreducens’ cells in a specific environment.

Among the different environments, ‘*Candidatus* M. nitroreducens’ was most abundant in rice field soil, followed by wastewater treatment plant sludge. The lowest copy numbers were obtained in the investigated river sediments (Fig. 2). In a previous study (Ding et al. 2015), 16S rRNA gene primers were designed to quantify ‘*Candidatus* Methanoperedens nitroreducens’ in two lake sediments, a river sediment, and a rice field soil sample. In that study, the total abundance of 16S rRNA gene copy numbers in rice field soil was one to two orders of magnitude lower than that obtained in the present study (3.72 × 10^4^ to 2.30 × 10^5^ copies μg^−1^ DNA versus 1.7 ± 0.4 × 10^6^ copies μg^−1^ DNA in this study). This variation may be due to differences in the environmental samples used; in addition, the 16S rRNA gene primers used in that study may have been more species-specific. Importantly, the relatively high gene copy numbers obtained in both studies suggest that these anaerobic methanotrophic archaea play a significant role in mediating nitrate-dependent AOM in rice fields and contribute to mitigating methane emissions to the atmosphere.

For accurate phylogenetic analysis, only a few ‘*Candidatus* M. nitroreducens’ *mcrA* gene sequences with lengths greater than 500 bp are available in public databases. These sequences were derived from deep groundwater (Nyyssonen et al. 2012), paddy fields (Bao et al. 2014), river sediments (Jiang et al. 2011), and lake sediments (GenBank accession number JQ080004, unpublished). All of these sequences were retrieved with the general *mcrA* primer pair ME1F/ME2R, which yields a sequence length of 763 bp (Hales et al. 1996). These primers have a high number of mismatches with the two available full-length ‘*Candidatus* M. nitroreducens’ *mcrA* sequences: six mismatches in the forward primer and five in the reverse primer. Thus, the presence of these microorganisms and their diversity in environmental studies may be underestimated because presently used primers simply do not capture them. These archaea have been assumed to be freshwater microorganisms, and thus, it is even more remarkable that we amplified both 16S rRNA and *mcrA* gene sequences of ‘*Candidatus* M. nitroreducens’ from marine North Sea sediment. The NCBI database contains only a few sequence entries from marine samples, e.g., accession number HM746653 (unpublished) and accession number GU182109 (Lever et al. 2013), which were detected in the sediment of the Gulf of Mexico and Juan de Fuca Ridge Flank basalt seafloor sediment, respectively. The sequences have 92 and 90% identity at the nucleotide level to the *mcrA* gene of ‘*Candidatus* M. nitroreducens’ (LKCM01000102.1), respectively. For comparison, the nitrite-dependent AOM bacterium ‘*Candidatus* M. oxyfera’ was reported in a recent study of the Eastern South Pacific oxygen minimum zone off Chile (Padilla et al. 2016). ‘*Candidatus* M. oxyfera’ had previously been solely linked to freshwater environments. However, it seems that both nitrite-dependent bacteria and nitrate-dependent archaea also have niches in marine ecosystems, and their roles in these environments remain to be elucidated.

In contrast to universal *mcrA* primers, universal 16S rRNA gene primers have successfully captured ‘*Candidatus* M. nitroreducens’ sequences with high identity to ‘*Candidatus* M. nitroreducens’ in several environments such as minerotrophic fens (Cadillo-Quiroz et al. 2008), river sediments (Li et al. 2012; Rastogi et al. 2009), lake sediments (Kadnikov et al. 2012; Schubert et al. 2011; Stein et al. 2001), contaminated soils (Kasai et al. 2005), groundwater (Flynn et al. 2013), mud volcanoes (Wrede et al. 2012), and Antarctic cold seeps (Niemann et al. 2009), among other environments. Based on 109 sequences of the GOM Arc I group in ARB, the phylogenetic trees not only show that the sequences of this phylogenetic group form a distinct cluster but also indicate that their diversity can be further divided into sub-branches within the cluster (Welte et al. 2016). This diversity is partially correlated with the environments from which the sequences were retrieved. Due to the lack of suitable primers, there are insufficiently high-quality *mcrA* sequences available to perform a similar analysis. This study added 28 long ‘*Candidatus* M. nitroreducens’ sequences (1191 bp) suitable for high-resolution phylogenetic analysis (Fig. 3). Additional sequences are needed to confirm the splitting of the *mcrA* gene diversity of ‘*Candidatus* M. nitroreducens’ into sub-branches. Furthermore, additional *mcrA* gene sequences will permit an investigation of the possible link between the phylogeny and distribution of ‘*Candidatus* M. nitroreducens’ in nature.

In this study, we designed two novel primer sets targeting the *mcrA* gene of the anaerobic methanotroph ‘*Candidatus* M. nitroreducens’: one set suitable for quantification and the other for detailed phylogeny. These molecular tools will enable the quantification and classification of these recently discovered anaerobic microorganisms in nature and, in turn, facilitate the further elucidation of the role of this important group of archaea in global nitrogen and methane cycling.

## Electronic supplementary material

ESM 1 (PDF 314 kb)
