# Supplementary material for: McrA primers for the detection and quantification of the anaerobic archaeal methanotroph ‘Candidatus Methanoperedens nitroreducens’
Source: Appl Microbiol Biotechnol. 2017 Jan 13;101(4):1631–41. doi: 10.1007/s00253-016-8065-8 (PMC5266762; doi:10.1007/s00253-016-8065-8)
Supplement: Supplementary file 1 — (PDF 314 kb) [file 253_2016_8065_MOESM1_ESM.pdf]

Applied Microbiology and Biotechnology

McrA primers for the detection and quantification of the anaerobic archaeal methanotroph '*Candidatus* Methanoperedens nitroreducens'

Annika Vaksmaa[1], Mike S. M. Jetten[1,2,3], Katharina F. Ettwig[1], Claudia Lüke[1]

[1]Department of Microbiology, IWWR, Radboud University Nijmegen, Nijmegen, the Netherlands

[2]Department of Biotechnology, Delft University of Technology, Delft, the Netherlands.

[3]Soehngen Institute of Anaerobic Microbiology, Nijmegen, the Netherlands

Running Head: McrA primers for 'Candidatus Methanoperedens nitroreducens'

Corresponding Author addresses (clueke@science.ru.nl, avaksmaa@science.ru.nl)

Supplementary Table S1: Description of the environmental samples and geographic location of sampling sites

Sample ID	Sampling location	Geographic coordinates
WWTP	Wastewater treatment plant sludge, Netherlands	51° 31´03´´N; 5° 36´41´´E
RF	Rice field soil, Italy	45°19’27’’N; 8°22’26´´E
NS	North Sea sediment, Netherlands	54˚13’00’’N; 4˚33’01’’ E
SC	State Channel sediment, USA	40°54'29’’N; 111°55'50’’W
JR	Jordan river sediment, USA	40°44'30’’N; 111°55'4’’W
IR	Citarum river sediment, Indonesia	6°56'30’’S; 107°32'10’’E

Supplementary Table S2: The mcrA clone sequence identities retrieved with the McrA159F/McrA345R primers from environmental samples and AOM enrichment culture. The identity on nucleotide level is compared to full length mcrA sequences of two '*Candidatus* M. nitroreducens' strains.

Environment	*mcrA* clone	Identity % to Methanoperedens nitroreducens (JMIY01000002.1)	Identity % to Candidatus Methanoperedens sp. DS-2015 (LKCM01000102.1)
WWTP	1	94	91
WWTP	2	95	92
WWTP	3	95	92
WWTP	4	93	90
WWTP	5	94	91
Jordan river	1	95	92
Jordan river	2	95	92
Jordan river	3	95	92
Jordan river	4	95	92
Jordan river	5	95	92
Rice field	1	95	94
Rice field	2	97	91
Rice field	3	94	94
Rice field	4	96	92

Rice field	5	95	94
AOM enrichment Vercelli	1	95	92
AOM enrichment Vercelli	2	95	92
AOM enrichment Vercelli	3	95	92
AOM enrichment Vercelli	4	100	92
AOM enrichment Vercelli	5	95	92
AOM enrichment Vercelli	6	95	92
AOM enrichment Vercelli	7	95	92
Indonesian River	1	99	92
Indonesian River	2	99	93
Indonesian River	3	99	93
Indonesian River	4	94	91
Indonesian River	5	94	91
Indonesian River	6	95	92
North Sea	1	94	91
North Sea	2	95	92
North Sea	3	95	92
North Sea	4	94	91
North Sea	5	95	92
State Channel	1	94	91
State Channel	2	94	91
State Channel	3	95	92
State Channel	4	95	92
State Channel	5	95	92
State Channel	6	94	91
State Channel	7	95	92

Supplementary Table S3: The 16S rRNA clone sequence identities retrieved with the AAA641F/AAA834R 16S rRNA gene primers from environmental samples and AOM enrichment culture. The identity on nucleotide level is compared to full length 16S rRNA gene sequences of two '*Candidatus* M. nitroreducens' strains.

Environment	16S rRNA clone	Identity % to Methanoperedens nitroreducens (JMIY01000002.1)	Identity % to Candidatus Methanoperedens sp. DS-2015 (LKCM01000080.1)
AOM enrichment Vercelli	1	99	99
AOM enrichment Vercelli	2	99	99
AOM enrichment Vercelli	3	100	99
North Sea	1	99	99
North Sea	2	100	99
North Sea	3	99	98
North Sea	4	99	99
North Sea	5	99	98
WWTP	1	99	98
Indonesian River	1	99	98
Indonesian River	2	99	99
Indonesian River	3	99	99
Indonesian River	4	99	99
Indonesian River	5	99	99
Indonesian River	6	99	99
Rice Field	1	99	99
Rice Field	2	99	99
Rice Field	3	99	98
Rice Field	4	99	98
Rice Field	5	99	99
Rice Field	6	99	99

Supplementary Table S4: The mcrA clone sequence identities retrieved with the McrA169F/McrA1360R primers from environmental samples and AOM enrichment culture. The identity on nucleotide level is compared to full length mcrA gene sequences of two '*Candidatus* M. nitroreducens' strains.

Environment	mcrA clone	Identity % to Methanoperedens nitroreducens (JMIY01000002.1)	Identity % to Candidatus Methanoperedens sp. DS-2015 (LKCM01000102.1)
WWTP	1	86	96
WWTP	2	86	94
WWTP	3	86	95
WWTP	4	86	94
Jordan river	1	86	96
Jordan river	2	85	95
Jordan river	3	86	96
Jordan river	4	85	95
Rice field	1	89	91
Rice field	2	86	98
Rice field	3	87	93
Rice field	4	87	98
AOM enrichment Vercelli	1	88	93
AOM enrichment Vercelli	2	88	89
AOM enrichment Vercelli	3	88	89
AOM enrichment Vercelli	4	86	96
Indonesian River	1	86	96
Indonesian River	2	87	91
Indonesian River	3	88	89
Indonesian River	4	88	91
North Sea	1	86	99
North Sea	2	86	99

North Sea	3	86	99
North Sea	4	87	95
State Channel	1	86	98
State Channel	2	86	94
State Channel	3	86	95
State Channel	4	86	95
